# Immunobiology of Cervix Ripening

**DOI:** 10.3389/fimmu.2019.03156

**Published:** 2020-01-24

**Authors:** Steven M. Yellon

**Affiliations:** Department of Basic Sciences, Longo Center for Perinatal Biology, School of Medicine, Loma Linda University, Loma Linda, CA, United States

**Keywords:** parturition, macrophage, inflammation, collagen, immune cells, extracellular matrix, preterm birth

## Abstract

The cervix is the essential gatekeeper for birth. Incomplete cervix remodeling contributes to problems with delivery at or post-term while preterm birth is a major factor in perinatal morbidity and mortality in newborns. Lack of cervix biopsies from women during the period preceding term or preterm birth have led to use of rodent models to advanced understanding of the mechanism for prepartum cervix remodeling. The critical transition from a soft cervix to a compliant prepartum lower uterine segment has only recently been recognized to occur in various mammalian species when progesterone in circulation is at or near the peak of pregnancy in preparation for birth. In rodents, characterization of ripening resembles an inflammatory process with a temporal coincidence of decreased density of cell nuclei, decline in cross-linked extracellular collagen, and increased presence of macrophages in the cervix. Although a role for inflammation in parturition and cervix remodeling is not a new concept, a comprehensive examination of literature in this review reveals that many conclusions are drawn from comparisons before and after ripening has occurred, not during the process. The present review focuses on essential phenotypes and functions of resident myeloid and possibly other immune cells to bridge the gap with evidence that specific biomarkers may assess the progress of ripening both at term and with preterm birth. Moreover, use of endpoints to determine the effectiveness of various therapeutic approaches to forestall remodeling and reduce risks for preterm birth, or facilitate ripening to promote parturition will improve the postpartum well-being of mothers and newborns.

The maternal immune system in mammals adapts to tolerate the differentiation of novel structures associated with the fetal allograft. Development of two interfaces protect the fetus from rejection and assault by the ecology of the external environment. The internal fetal-maternal interface, as represented by the fetal membranes, placenta, and decidua, is crucial for maintaining maternal inflammatory reactivity for surveillance and responses to pathogens. Discussion of the internal interface is elsewhere and in this special volume (1–3). However, the present review is focused on the external fetal-maternal interface consisting of an amniotic fluid buffer, in a forebag region later in pregnancy as the fetal head engages the lower uterus, and the fetal membranes as they press against the internal os of the cervix. This description of a singleton pregnancy in primates near term applies to rodents, despite anatomical differences in the uterus described below, because observations indicate a single fetal sac engages the internal os of the cervix shortly before labor. The success of this external interface to fend off the biome and virome in the vagina reflects the barrier function of the cervix to protect both the fetus and maternal host structures in the uterus. The cervix barrier function has both immunological and structural components. One part of the maternal immune external interface is the mucus-epithelial lining along the exterior of the ectocervix and lumen from the external to internal os. This immunological microenvironment was the focus of a recent review in non-pregnant women, but studies have yet to extend to pregnancy or the prepartum period for cervix remodeling (4). Further consideration of this part of the external interface is beyond the scope of this review. The second component of cervix immunobiology is the intimate relationship of immune cells with extracellular matrix collagen and fibroblasts that regulate structure. Thus, removal of the structural obstacle for parturition reflects the second function of the cervix—to virtually disappear for birth, and the primary focus of this review.

The overall objective of this review is to advance appreciation that the cervix is a uniquely distinct structure and timing of remodeling occurs in advance of labor. Analogous to an inflammatory process, evidence suggests that cervix remodeling before term involves a coordination of immune cell activities for degradation of collagen structure in the extracellular matrix. Understanding cervix anatomy and heterogeneity is critical to appreciate the context of data and their interpretations in the literature. The goals of this review are to update the current perspective about characteristics associated with structural changes cervix functions that occur well before the shift in contractile activity by the uterus for labor. These characteristics with respect to phases of remodeling are conceived as the end result of activities by resident immune cells—effectors of the physiological inflammation that is a non-scarring reversible process for the next pregnancy. The importance of cross-linked collagen alignment and essential requirement for sufficient disintegration of cervix structure in the stroma to eliminate the barrier for birth before other reproductive organs of pregnancy is emphasized because if the gate does not open, delivery does not occur. Evidence indicates that a balance of endocrine and immune cells activities may sustain the barrier function of the cervix while increased presence and functional activities by resident immune cells appear critical to eliminate the barrier for birth. Insights about the remodeling mechanism at term and identification of gaps are useful considerations for understanding advanced remodeling or an incompetent barrier for the pathophysiology of preterm labor. What little is known about incomplete cervix remodeling that leads to medical intervention for delivery is also discussed. Literature indicates that an improved understanding of cervical remodeling and its timing may lead to the development of novel approaches to treat cervical insufficiency or lack of compliance. Identifying biomarkers for remodeling and the potential for non-invasive imaging to assess their change with the progress of pregnancy have promise, in the case of PTB, to serve as sentinels for women at risk of preterm cervix incompetence. Assessment of biomarkers for advanced or delayed loss of the barrier to birth would gauge the efficacy of novel approaches to treat women at risk for preterm birth or for insufficient remodeling at term to avert the necessity of medical intervention to protect newborn and maternal well-being.

## Cervix Anatomy and Function for Pregnancy In Mammals at the External Fetal-Maternal Interface

Among placental mammals, the gross anatomy of the reproductive tract has long been recognized to be diverse (5). In some species, the uterus has two distinct horns with a septum and separate exits into a transition region (rabbits and rodents), while others converge into a uterine body (mares). For primates, the uterus is a simple singular structure. Such diversity does not extend to the cervix. Irrespective of the number of entrances from the uterus, a common canal from the internal to external os opens into the vagina. This cylindrical canal is surrounded by the endocervix toward the uterus then the ectocervix that protrudes into the vagina. Attachment of the vagina to the outer cervix distinguishes between these subregions. By gross visual inspection, it is difficult to identify the border between the uterus and cervix because the transition zone, aka uterine isthmus, can only be defined by histological presence or absence of uterine structures, i.e., endometrial glands, as well as circular and/or longitudinal smooth muscle. Moreover, common use of the term “uterine cervix” continues a misperception that the cervix is a “small portion of the uterus” (6) and diminishes the morphological distinction from the uterus. Leppert also points out that it is difficult to compare results from various laboratories, because biopsy sites are rarely described in detail. In many biochemical studies, biopsy tissue taken from the lower uterine segment after cesarean section delivery may not with certainty be “cervix,” i.e., no distinction of upper transition from uterus from what was endocervix before remodeling or dilation. Thus, differences in anatomy, function, and immunology of the uterus and other reproductive structures among mammals or conclusions about findings from studies of the lower uterine segment may not necessarily be relevant for the cervix or apply to the remodeling process for parturition.

**Figure 1:**
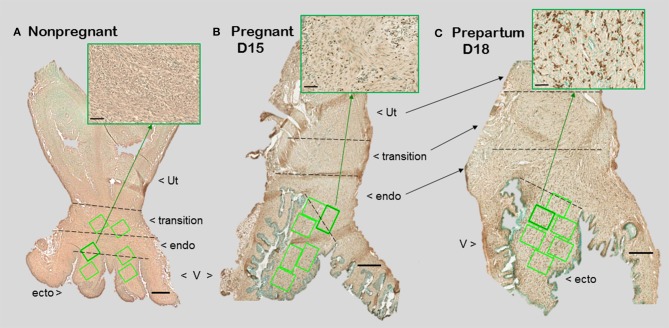
Photomicrographs of cervix section from mice that were **(A)** non-pregnant in estrus, **(B)** pregnancy day 15 (D15), 4 days before expected birth, or **(C)** prepartum day 18 of pregnancy (D18), the day before expected birth. Macrophages were immunostained brown with the F4/80 antibody while cell nuclei were counterstained with methyl green. Ut, uterus; V, vagina. Cervix subregions are ecto = ectocervix, endo = endocervix, transition = region before appearance of smooth muscle or glands of uterus. Scale bar is 500 μm or 50 μm for inset (zoom of area indicated by green arrow). Macrophages and cell nuclei are typically counted in a survey of 6–8 areas (green boxes) in each of 2 sections/mouse (1.5–2 × 10^6^ μm^2^).

With appreciation that the cervix is a distinct component of the reproductive tract, do gross morphological commonalities extend to the cellular level among mammals? Compared to the uterus, the cervix is highly innervated, more so at term (7, 8). During early development, the cervix differentiate from Müllerian duct mesenchyme under the influence of apposing ectoderm that ultimately forms the vagina (9, 10). Across species, the cervix consists of blood vessels, as well as fibroblasts, smooth muscle, and luminal epithelium, but the distinctive characteristic is a dense and heterogeneous extracellular matrix structure. This has been well-documented in non-pregnant women (11). For lymph flow out of the cervix, the supraureteral route is predominant among several pathways with multiple lymphatic nodes (12). No evidence supports a direct drainage of lymph from any region of the cervix to the uterus. Studies of lymphatic drainage have not been conducted during pregnancy or in other species. However, during pregnancy, cervix size increases, more in volume and possibly width than length in women (13, 14). In mice, cervix length is increased with fewer cell nuclei/area evident in stroma with pregnancy (Figure 1). A similar finding is evident in women before pregnancy compared to those pregnant whether or not in labor (15). Reduced cell nuclei density may reflect hypertrophy of cells or an increase in extracellular space. The majority of these cells are likely to be fibroblasts based upon morphology though no marker has been used to exclusively identify this cell type in the cervix.

Transformation of the barrier function of the cervix from a firm and relatively non-compliant structure before pregnancy occurs as pregnancy progresses and the contents of the uterus grows. Eventually, the barrier for birth must be removed. Evidence suggests that mechanical properties of the cervix dramatically change in direct relation to alterations in the dense network of cross-linked collagen from the mid-point of pregnancy to term compared to the non-pregnant state (11, 16, 17). To explain greater compliance and dispensability during the period leading up to term, studies have focused on collagen—the principal protein in the cervix among various species. Collagen type I is estimated to constitute as much as 70% of the fibrous extracellular matrix in the cervix (18). Collagen type III contributes >30% or less to the total. A small amount of collagen type IV associated with the vasculature and smooth muscle cells (19). By immunofluorescence, a decline in collagen type I was observed in biopsies of ectocervix from women in the 3rd compared to 1st trimester (20). Whether a change in the ratio of collagen types I to III in the cervix is related to compliance or remodeling throughout pregnancy is not known. Conclusions about total collagen in the cervix may vary. Reports in women (13, 18) and other mammals find that collagen concentration/wet weight of tissue declines as pregnancy progresses to term (21–25). By contrast, other studies indicate that collagen content/dry weight does not significantly change during pregnancy in both human (18, 26) and mouse tissue (27). Although the advantage for a dry weight analysis of total collagen content is not clear, more soluble collagen/dry weight was extracted from biopsies of cervix from pregnant women at term vs. those not pregnant. Solubility of collagen for extraction is an indication of immature, less cross-linked fibers.

For pregnant mice 4 days before birth, the cervix content of soluble collagen is increased as molecules associated with cross-linked collagen declined compared to that before pregnancy (27, 28). In particular, hydroxyproline is a major component of cross-linked collagen that is well-correlated with reduced cervix tensile strength. Evidence indicates that hydroxyproline content declines from about mid- pregnancy to term. Of critical relevance for remodeling is that hydroxyproline is lower in the cervix of preterm women not in labor (average gestation 30 weeks) vs. that in non-pregnant women (15, 29). Similarly by 10–12 days of pregnancy in mice, hydroxyproline is reduced compared to that in non-pregnants (30). Loss of insoluble cross-linked collagen was further indicated to be essential for softening and ripening of the cervix in a study of knock out mice with impaired ability to degradation this protein (31). Other common constituents of the cervix are hyaluronan, elastin, and proteoglycans (glycosaminoglycans). The importance of hyaluronan to cervix physiology and functions across species related to parturition and preterm birth was part of a recent review (32). However, the importance of glycosaminoglycans for cervix remodeling is not clear. This component of the extracellular collagen matrix declines in the cervix before term in sheep and women (13, 24). In addition, mice lacking decorin, the principal component of glycosaminoglycan, have little or no defects in the timing or the process of parturition (33). Only when both the proteoglycans decorin and biglycan are absent in mice does preterm birth ensue. The effects of this double knockout model on the cervix have yet to be studies. Collectively, these findings focus attention on the reduction in cross-linked collagen and hyaluronan as essential for cervix remodeling.

Subregional heterogeneity of the cervix has been recognized. More than 60 years ago a study of serial cross-sections from the distal ectocervix through endocervix of the rat by Harkness et al. found that collagen content and cross-linking declined in from the external to internal os (21). This conclusion has since been confirmed in both rats (6) and humans (11). The relative ratio of collagen and connective tissue to smooth muscle also varies along the length of the cervix- greater in the distal region than the cervix area closer to the myometrium. In mice 4 days before birth (day 15 of a typical 19 day pregnancy), no differences in morphological or cross-linked structural characteristics were found in analysis of the ectocervix, endocervix, or transition zone (34). However, such a subregional analysis has yet to be done during the period leading up to birth at term in any species. In women, ultrasound analyses indicates variations in alignment characteristics in the circumferential and longitudinal axes along the length of the cervix during pregnancy (35). These differences were incorporated into a model that predicted variations in mechanical responses within subregions of the cervix as pregnancy progresses to term. Second harmonic generation analyses of cervix from non-pregnant women found circumferential and subregional differences in collagen alignment (36). Trends for less-alignment in the cervix from women with abnormal placentation at term compared to that in contemporary controls require further study. Comparable analyses have yet to extend to non-human primates or other species. Moreover, further study using non-invasive image analyses would strengthen the accepted conclusion that reduced alignment and less anisotropy, as determined by, directly reflect a decline in extracellular cross-linked mature collagen with remodeling. By example, photoacoustic imaging was recently used to identify that increased water content accompanied collagen disorganization in the ripened vs. unripe cervix (37). The relationship of edema, cellular hypertrophy, cell nuclei density, and cross-linked collagen in the stroma could clarify if heterogeneity within and along subregions may be extrapolated from a biopsy of one area to a broader conclusion about the entire cervix. Even so, the current status of cross-species comparisons suggests substantial similarities between humans and rodent models in fundamental characteristics of the cervix during pregnancy (summarized in Table 1). This comparison provides support for use of rodent models to advance understanding of prepartum remodeling and to develop hypotheses, as well as potential biomarkers for studies of the shift from a soft to ripe cervix in primates.

**Table 1:** Common characteristics of prepartum cervix remodeling in women and rodents at or near term vs. earlier or before pregnancy.

**Characteristic**	**Human (term)**	**Mouse/Rat (term)**
Collagen structure (OD, optical density)	↓ TNL (15)	↓ (38, 39)
Macrophages (vs. NP)	↑ (40)	↑ (39)
Innervation	Abundant (7)	↑ and abundant (8)
Pro-inflamm. cytokines	↑ (41)	↑ (42)
PR antagonist effects- preterm birth (RU486/Onapristone)	Ripens (43–46) ↓ CN TNL v NP (15)	Ripens ↓ Structure (OD collagen) ↓CN, ↑Mφ (39)
Systemic P4	↑ to PP (47, 48)	↑ to <2 days of birth (48)
Systemic E2/P4	↑ (49)	↑ (50)

*↓, decrease; ↑, increase; TNL, term no labor; (#), reference number; E2, estradiol, P4, progesterone; CN, cell nuclei; Mφ, macrophages; NP, nonpregnant; RU486, mifepristone; PP, postpartum*.

Biomolecular studies of collagen in dispersed cervix are a common approach to study remodeling. The disintegration of structure to study cells and molecules present other challenges that do not take into consideration subregional tissue heterogeneity or changes in morphology as pregnancy progresses to term. As already mentioned above, biopsy tissue are unlikely to accurately characterize the entire human cervix. For animal models, studies rarely provide details about what defines an excised cervix. This is important, as exemplified in a study in mice, in which a wide range of cervix weights/day of pregnancy was reported and may reflect a varying presence of adherent vaginal and/or uterine tissue remnants (38). Moreover, accurate quantification depends upon clear anatomical boundaries, which is not possible by gross dissection given the intimate attachment of the vagina at the ectocervix-endocervix boundary and, as specifically mentioned by Harkness et al. (21), in the transition zone into the uterus (see Figure 1). For collagen analysis, Masson's trichrome stain is commonly used in various tissues and for the cervix during pregnancy. However, Masson's stains non-collagenous structures, as well as collagen and smooth muscle (51, 52). Differentiation of pink/blue transitions by observations or image analyses are subjective, may vary among sections, and rarely quantified. Of importance for the cervix, the area of Masson's stain is not informative of the major change in cross-linked collagen structure or heterogeneous distribution within subregions from the external to internal os, aspects of which seem critical for understanding cervix remodeling. Moreover, stromal collagen degradation as a biomarker for cervix remodeling has not been linked with indices of cervical epithelial barrier integrity, such as mucin or E cadherin, which have served as sentinels for vaginal inflammation (53).

**Figure 2:**
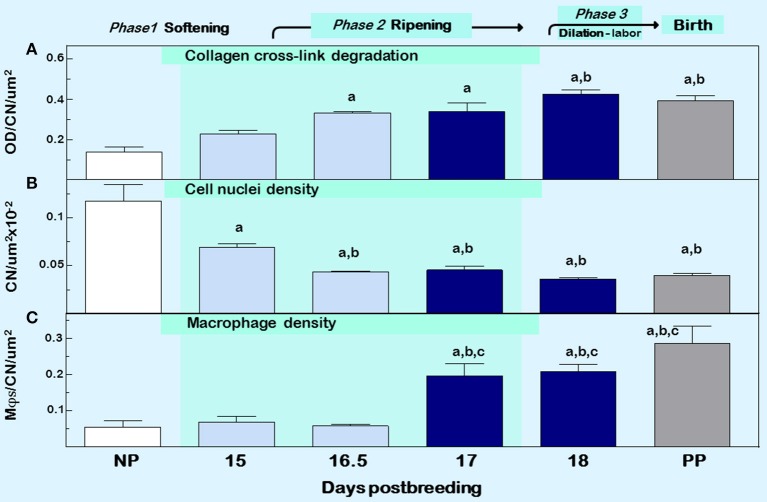
Cervix ripening between days 15 and 18 of pregnancy in mice. Characteristics associated with cervix remodeling in CD-1 mice during days postbreeding of pregnancy and postpartum on day 19 postbreeding [derived from (39)]. **(A)** Optical density (OD; mean + SEM; *n* = 3–10) of polarized light from birefringence of picrosirius red–stained sections. OD was calculated using the Rodbard transformation of NIH Image J is inversely related to birefringence. This approach provides a quantitative assessment of cross-linked collagen structure which when OD is increased reflects greater light transmittance, an indication of collagen fiber disarray, decline in cross-linking, reduced length, and smaller diameter in the extracellular matrix (55). **(B)** Cell nuclei (CN) density, and **(C)** density of macrophage (Mϕs). Data for OD and Mϕs were normalized to cell nuclei density/section for each mouse to account for variability in the area of extracellular space, cell size, cell numbers, and morphology across sections, individuals, and groups. Area for Mϕ and CN density analyses averaged is 1.251 × 105 μm^2^/mouse cervix. Data are expressed as mean ±SE (*n* = 4–6; ANOVA with Dunnett test). ^a^*p* < 0.05 vs. NP, ^b^*p* < 0.05 vs. D15, ^c^*p* < 0.05 vs. D16.5.

Another approach to assess collagen in the extracellular matrix is staining with the dye picrosirius red. Two reviews summarize the usefulness of this stain over the past 40 years and its application to quantify cross-linked collagen (54, 55). The latter paper reviewed findings that reflect misaligned and disorganized fibers due to lack of collagen cross-linking in the cervix stroma of multiple strains of mice and rats with treatments that advance or forestall birth, as well as in peripartum women both preterm and at term. The method is not based upon intensity or area of stain in tissue sections, which may vary with processing or section conditions, region of section analyzed, or reproductive status. Rather, optical density of birefringence of polarized light from picrosirius red stained cervix is inversely and specifically related to reported declines in cross-link collagen with respect to hydroxyproline. This conclusion was also supported by electron microscopic analyses of collagen fibril diameter and length in rodents (30, 56, 57). In a survey of picrosirius-stained sections that focused on the stroma from ectocervix through endocervix in mice (39), findings provided the first indication that degradation of cross-linked collagen occurred much earlier than previously thought, more than 3 days before birth (Figure 2, top panel). This approach was also used to study degradation of collagen in HSV2 infection-induced cervix ripening and preterm birth (58). Thus, well before term, characteristics of extracellular matrix remodeling was evident. As a percentage of pregnancy, this period of gestation is chronologically analogous to the period between 32 and 37 weeks of pregnancy in women and acknowledged in clinical reports described below to coincide with remodeling changes in cervix structure.

Other sentinels that reflect the transformation in cervix morphology characterize the process of inflammation, generally defined by swelling, increased presence of immune cells, and loss of function (4). Specifically as shown in Figure 2 middle and lower panels, reduced cell nuclei density attests to fewer or larger cells/area, possibly an expansion of extracellular space or cell growth (39). In addition, increased census of macrophages/area of cervix before term reflects a local source for collagenases, metalloproteinases (MMPs), proinflammatory cytokines, nitric oxide, and prostaglandins that regulate extracellular matrix structure (59). The traditional view that bone marrow-derived monocytes are the immediate precursors of tissue macrophages needs to be reexamined based upon evidence that macrophages in tissue can extensively self-renew and be seeded from yolk sac/fetal liver progenitors with little input from circulating monocytes (60). In humans, reserve/basal cells at the endo–ectocervix junction may be progenitors for squamous and/or columnar epithelium in the endocervix (61). Whether macrophages in the cervix during pregnancy are recruited and differentiate from precursors, either systemic monocytes or resident stem cells, has yet to be studied. Moreover, the third component of swelling from inflammation is loss of function—clearly indicated by degradation of collagen structure and greater compliance that eliminates the barrier for birth during prepartum remodeling (described above). These morphological features characterize prepartum cervix remodeling several days before labor, which is estimated to begin, as assessed by an increase in uterus mRNA levels for contraction-associated proteins, by the afternoon of the day before birth within 19 h of appearance of the first pup in mice (62). As detailed in the next section, these concepts leads to appreciation that remodeling reflects a physiological inflammation in the prepartum cervix as part of a precisely timed sequence that allows for dilation and vaginal birth.

## Immunobiology Associated With Phases of Cervix Remodeling

Clinical observations from studies that primarily focused on uterine contractile activity provided clues about remodeling of the cervix with the progression of pregnancy. In particular, Caldeyro and Poseiro noticed that the cervix softens then ripens during a period known called as prelabor when uterine activity begins to increase sometime after 30 weeks of pregnancy (63). The process of cervical ripening, as characterized by softening, effacement, and eventually early signs of dilatation was observed to begin about 4–6 weeks before birth (64). In a well-reasoned review, Kelly mentioned that Liggins advanced the idea that softening and dilatation, a so-called ripening of the cervix, was a “poorly understood inflammatory process” (65). Based upon a variety of species, available data was then used to suggest that proinflammatory activities by specific chemokines, cytokines, and prostaglandins in amniotic fluid, perhaps related to fetal membranes, decidua, or in the cervix itself, soften the internal os of the cervix. In a later review, Kelly discussed loss of the barrier function of the cervix in terms of actions by neutrophils, prostaglandins, and nitric oxide (66). Also featured was the importance of the cervix barrier to prevent ascending infection by mucus secretion and active innate immune defenses at the epithelial surface along the ectocervix and endocervix. The cervix thus functions to keep the fetus *in utero* during softening and, after ripening, allowed for dilatation at birth. Though focus of these reviews were upon the mechanism to ripen the cervix, there was limited morphological, cellular, or biomolecular information to define the terms “softening” or “dilation” or to characterize what “ripening” actually was from start to finish. Rather, conclusions to suggest that ripening may result from increased IL-8 related to ingress of neutrophils and their release of collagenases, MMP-8, and MMP-9 were based, to a large part, upon analyses of the lower uterine segment from women at term after cesarean section (67). In retrospect, this understanding of ripening is complicated by the fact that information came from biopsies of women that required medical intervention for delivery. In context, these state-of-the-art reviews occurred when the Bishop score, a commonly used Obstetrical practice to subjectively assess cervix preparedness for induction of labor in multigravida women at term, was being repurposed as an indicator of ripening during pregnancy (68–70).

To clarify endpoints that define transitions in cervix remodeling, advances from studies of collagen and related enzyme activities led Word et al. to adapt concepts in a previous figure (63) to elucidate 4 distinct phases of cervix remodeling—softening, ripening, dilation, and repair of the cervix (71). Cervical ripening was defined as “increased softening, decreased rigidity, effacement, and early dilation.” Concurrently, another review from this group summarized the importance of inflammatory cells and expression by related genes during softening of the cervix in mice (50). Without qualifications or explicit definition of phase-specific remodeling characteristics, the period of cervix softening expanded as a percentage of pregnancy while the time for ripening was compressed to >20 h on the day before birth. In context, each of these reviews provided the best insights from primary reports about endpoints associated with remodeling. Biomarkers to clearly distinguish these phases were unavailable. Little was known about immune cells in the cervix of women before 38 weeks of pregnancy, except for a study of ectocervix biopsies in women during the first trimester where macrophages were found to be sparse (72). Complimenting these efforts were studies in rodents, which provided support for inflammation as intrinsic to the ripening process. The discovery that macrophages were increased several fold by the day before birth in mice compared to earlier in pregnancy (73) suggested this immune cell helped to maintain local immune activities. With hindsight, available literature lacked longitudinal and cross-sectional time course data, both in women as expected because of limited access to biopsy tissues, but also for all animal models as pregnancy neared term. In fact, most rodent studies, including from my lab, compared biomolecular characteristics of the cervix from 3 or more days before birth to those on the day before birth (day 18–19 postbreeding). From these data, conclusions were extrapolated about ripening during the intervening period. By example, a review (31) concluded that the softening phase of remodeling is associated with a decline in collagen cross-link density, an increase in water content, and no significant changes in the glycosaminoglycan content based upon comparison of results in the cervix of mice on day 12 of pregnancy to non-pregnants (30). For the ripening phase, a rapid decrease in collagen cross-linked density occurred by day 18 of pregnancy, the day before delivery, compared to day 15 of pregnancy (74). During this period, water content increased while hyaluronan content doubled. However, groups were not studied between non-pregnant and days 12 or 18 of pregnancy. As discussed below, most studies and reviews prior to 2015-based conclusions about softening and ripening without sufficiently frequent time points to resolve when one phase concluded and another began. Comparisons of data sets across species were often cites to bridge gaps. Realistically, conclusions from these studies and reviews were deduced from comparisons of data from groups before the start of vs. after the finish of ripening. With this new perspective, it seems reasonable to revisit the question of whether inflammation drives phases of remodeling in preparation for birth at term.

### Softening

The study of immune cells in circulation in circulation before 36 weeks of pregnancy is indicative of suppression of some adaptive immune activities while parts of the innate immune system are activated (75). With limited availability of cervix biopsies in women between 30 and 38 weeks of pregnancy, a non-pregnant mouse model for pregnancy was developed to investigate morphological and immune cell characteristics associated with softening of the cervix and the role of sex steroids (76). Specifically, the decline in cell nuclei density and degradation of cross-linked collagen (optical density of birefringence of polarized light) in the cervix following estradiol and progesterone treatments of non-pregnant mice for 15 or 18 days were directly comparable to that found on day 15 or 18 of pregnancy. For ripening, removal of only the progesterone capsule on treatment day 17, to mimic withdrawal in circulation on days 18–19 of pregnancy, increased the presence of macrophages, and neutrophils in the cervix-comparable to results in pregnant mice at term. Neither cell nuclei nor collagen optical density was affected by progesterone withdrawal. These findings suggest that sex steroids may regulate morphological characteristics of the cervix for softening and the increased presence of immune cells associated with ripening. This contention is further supported in subsequent studies of progesterone receptor (PR) antagonist-induced advance in preterm cervix remodeling, as well as in PR agonist forestalled preterm parturition (39).

### Ripening

The American College of Obstetricians and Gynecologist defined cervical ripening as the prelude that occurs weeks before labor use (61, 77). In circulation at term, increased proinflammatory myeloid and lymphoid phenotypes, related cytokines, regulators of chemotaxis, and the capability to induce oxidative stressors may be key components of the parturition process at term and, if inappropriately advanced, may contribute to processes that lead to preterm birth (78–80). As apparent in mice (Figure 1 insets and 2), changes in characteristics of ripening in mice occurs 2–4 days before birth (days 15–17 postbreeding) when progesterone is near or at peak concentrations in circulation (39, 47). These findings in 2 rodent species and a variety of strains, as well as in women (15), indicate that structural changes in the stroma, i.e., reduced cell nuclei density and degradation of cross-linked collagen while progesterone in circulation is elevated, occur well before the uterus develops contractile capabilities for labor at term (62). The apparent withdrawal of progesterone efficacy in rodents and humans for cervix ripening, does not contradict clear evidence that the decline in serum progesterone is likely to be important for labor in rodents and other species compared to sustained high concentrations in circulation in primates throughout pregnancy (81, 82). Perhaps the more important insight from these studies in rodents and from early reviews of cervix remodeling in women is that the ripening phase occurs well before term and is distinct from the dilation phase, which is associated with dilatability in response to labor. With this perspective, immune cells in the cervix of women at term whether or not in labor may be considered part of the prepartum prelude to the dilation phase of remodeling.

For macrophage, a greater presence during the ripening suggests their participation in a local inflammatory process. In mice, increased residency by macrophages occurs in the prepartum cervix stroma by day before birth compared to earlier in pregnancy in several studies using different strains [(83, 84); shown in Figure 1 insets]. This conclusion was replicated and macrophages found to increase in the cervix by day 17 of pregnancy in a later investigation [Figure 2 derived from (39)]. Moreover, a recent study used a conditional knockout mouse model to deplete CD11b^+^F4/80^+^ macrophages throughout various subregions early in the ripening phase. As the first study to histologically confirm macrophage depletion during the transition from the soft to ripening phase of pregnancy, evidence supported a role for this immune cell in remodeling (34). The lack of collagen cross-linked degradation coupled with reduced cell nuclei density in the stroma when macrophages were depleted suggested ripening was blocked, but inflammation was ongoing due to extracellular space expansion or hypertrophy of cells. These effects on remodeling were overshadowed by evidence that treatment caused fetal demise without preterm birth possible through impaired placental function led. Finally, a study of cervix biopsies from around 30 weeks of pregnancy reinforces the conclusion that resident macrophages were increased compared to that in women at term not in labor (15). The collective implication is that morphological evidence has accumulated to reinforce the concept that the presence and likely activity of macrophages are an essential part of the process that remodels the cervix in preparation for parturition.

Recognition that macrophages are important for ripening, leads to specific questions about their function for remodeling. Flow cytometry has identified molecules expressed by immune cell phenotypes associated with functional activities. This approach was used to study potential macrophage functions in the cervix as pregnancy nears term (85). In this study, stringent criteria to eliminate systemic blood before dispersion of cervix and use dispersed spleen as a control to set gates were necessary to focus on living resident immune cells. Findings confirmed increased macrophages in the prepartum cervix by the day before vs. 4 days before birth. In addition, markers expressed by macrophages for MMP activation (CD147) and cell matrix remodeling (CD169) are present in the cervix on day 18 vs. day 15 of pregnancy. A reduced presence of macrophages with markers associated with adhesion (CD11b^high^) and migration (CD54) were found on the day prior to birth (day 18 postbreeding) than in mid/late gestation (day of pregnancy). These results suggest that activities by cervix macrophages are probably not related to migration, but remodeling and extracellular matrix degradation, which are important processes associated with increased biomechanical compliance in preparation for dilation and effective labor as previously described. Although tissue preparation and data analyses may account for differences in some findings (86), further investigations are needed at more times during the transition from soft to ripening to understand the complex balance of local activities by macrophages.

A comprehensive review of immune cells at the internal fetal-maternal interface in humans and rodents recognized inflammation as a central component of the mechanism of labor (42). Though less information is available for the external fetal maternal interface that involves the cervix, evidence clearly indicates increase in presence of macrophages in the cervix before the day of birth in rodents (83–85, 87). However, further study in mice during the transition from a soft to ripening cervix now indicates inflammation occurs earlier, between day 15–17 of pregnancy, with respect to reduced densities of cell nuclei and cross-linked collagen and greater abundance of resident macrophages with proinflammatory phenotypes (Figure 2). The temporal relationship between these remodeling changes in the cervix stroma, at the external fetal-maternal interface, and inflammatory processes in the decidua and placenta remains to be determined.

Macrophages are not the only myeloid-derived immune cell in the cervix during pregnancy though information about phenotypes and activities vary widely across species. Dendritic cells, a myeloid-derived antigen-presenting cell type, were sparse or not found in cervix biopsies from non-pregnant women (88, 89), but abundant in the peripartum period (90). For other polymorphonuclear leukocytes, neutrophils in particular, their increased presence in peripartum vs. non-pregnant cervix of women suggested a role in the ripening process and degradation of the extracellular collagen matrix (72, 91–93). Further study has not supported this conclusion because a greater census of neutrophils in the cervix occurs only near labor or after degradation of collagen becomes evident in women and rodents (39, 81, 84, 94–96). For eosinophils, there are more than a few similarities in morphology and functions for this immune cell in humans and mice (97). In women, degranulated eosinophils were abundant in the cervix from postpartum women, but not in those whom were pregnant or non-pregnant (98). Similarly in rats, more eosinophils were present on the day of birth (99). In cows, no changes in resident neutrophils and eosinophils were found in the cervix on day 185 vs. 275 of pregnancy, about 95 and 5 days before birth (100). For mast cells, numbers and activity were increase in ectocervix biopsies from women at term compared to that in the first trimester of in non-pregnants (101). In the cervix stroma in rats, numbers of eosinophils are reported to decline with the conclusion of pregnancy, their proportion increases by the day of birth (102, 103). Although further study of other myeloid cells as pregnancy progressed to term is needed, these findings do not support a role for neutrophils, eosinophils, or mast cells in the softening or ripening phases of cervix remodeling. The possibility remains that these cells may contribute to prepartum dilatation or peripartum processes that repair the cervix for postpartum restoration of barrier functions.

Lymphoid-derived immune cells are also present in the cervix of humans and rodents. Both T helper lymphocytes (CD4^+^) that respond to infection as part of the adaptive immune system, and cytotoxic T cells (CD8^+^) that surveil for virus-infected or damaged cells, are present in cervix biopsies throughout the menstrual cycle from non-pregnant women (89). Mostly located in the subepithelial stroma of the ectocervix and transition zone (isthmus), CD4+ lymphocytes were more abundant that CD8+ cell. B lymphocytes were scarce and predominantly in the suprabasal and subepithelial layers, but not stroma of the cervix. No differences were evident in number or distribution of immune cells between proliferative and secretory phases of the cycle. In ectocervix biopsies during the first trimester of pregnancy, T lymphocytes constituted half of all leukocytes and most were CD8+ (72). B lymphocytes were present in low numbers. By comparison, leukocytes were increased 2-fold in the cervix at term with an increase of 4-fold in CD4+ T cells in women not in labor and 10-fold in women during labor. Given the long gap in available tissue biopsies between first trimester and term, no study has focused on the temporal relationship between resident immune cells and degradation of cross-linked collagen during the softening or ripening phases of cervix remodeling. However, during cervical dilatation, a massive leukocyte presence if found in the cervix stroma compared to that in non-pregnant women (104). In mice, flow cytometry analysis of dispersed cervix on D18.5 of pregnancy indicated no difference in density of CD4^+^ T cells vs. that in non-pregnants (105). Overall conclusion about the role of lymphoid-derived cells in cervix function await further study at more frequent time points relative to phases of remodeling. As part of the approach to this effort, an situ histological approach is needed to distinguish T cell phenotypes that may contribute to mucosal-epithelial immune functions compared to lymphocytes in the stroma that may regulate changes in extracellular matrix structure. Distinguishing between T cell functions related to immune surveillance or possible antigen presentation actions that may locally activate T cells and secrete cytokines could help explain why activation of maternal T cells induces preterm birth in mice (106). Though the cervix was not a focus of study, activation of the T cell CD3ε receptor is presumed to induce ripening in advance of preterm birth in this model.

Dilation is the part of remodeling when the cervix opens in preparation for birth. Opening of the gate for birth is the ability of the cervix to accommodate passage of a newborn. Rather than the process of ripening, the capability to fully dilate may define completion of the phase of ripening. As previously mentioned, this phase of remodeling is characterized by pre-labor and the progress of contractions by the uterus. In many respects, the phase of dilation has been well-studied in women due to the availability of cervix biopsies at term whether or not in labor as well as with induction of labor at term. For decades, the Norman group has pioneered studies of the inflammatory process in reproductive tract and the cervix in particular at term (95, 107–110). With a focus on the cervix stroma, the densities of macrophages and neutrophils per area, not T (CD3+ cells) or B lymphocytes, as well as message for related proinflammatory cytokines (IL-1β, IL-6, IL-8, and TNFα) were greater in laboring vs. not in labor women. Increased concentrations of granulocyte-macrophage colony stimulating factor in the cervix during labor may also stimulate growth and activity of resident myeloid cells (94). Leukocytes are considered the main source of these cytokines. In another study, resident macrophages were also found to increase in the cervix, both in stroma and subepithelial regions, in women at term compared to that about 30 weeks of pregnancy (15). Though there were no differences in macrophage density in laboring vs. non-laboring women at term, degradation of cross-linked collagen and reduced cell nuclei density were found compared to that in cervix biopsies earlier in pregnancy. These structural changes may result from the actions of proinflammatory cytokines that promote proteinase digestion of the extracellular matrix (111). Thus, at term, characteristics of inflammation were evident during the dilation phase of cervix remodeling. This conclusion is supported by increased presence of inducible nitric oxide synthetase (iNOS) in the cervix stroma of women at term, whether or not in labor (66). The coincident increase iNOS protein and number of resident leukocytes (107) provides a mechanism for vasodilation in order to facilitate leukocyte trafficking and the challenges posed to maintain tissue perfusion during compression associated with contractions of labor. Further understanding of this transition would benefit from investigation of the relationship of cervix dilation, i.e., opening of the external os, to uterine contractile activity and biomechanical properties, biomolecular concentrations of mature cross-linked collagen or related molecules, and proinflammatory biomarkers in the cervix at term during the progression to vaginal delivery.

Recovery during phase 4 of remodeling is essential for restoration of the barrier function of the cervix. This non-scaring process occurs rapidly in rodents within 2 days of birth in preparation for a postpartum estrus and the next pregnancy (112). Expression of message for molecules in the cervix during this period are associated with inflammatory and wound-healing functions. This process has not been studied in women and whether aspects of reconstruction of non-pregnant cervix structure begins prior to birth is not known.

## Functionality of Resident Immune Cells in the Prepartum Cervix

The presence of inflammatory cells during various phases of prepartum cervix remodeling provides the potential for local paracrine factors (cytokines and chemokines) to guide changes in extracellular matrix structure. Heterogeneity in cervix morphology within subregions may extend to distribution or density of immune cell phenotypes. As alluded to in a flow cytometry study of macrophages in the cervix of pregnant mice (85), analyses of cells from a dispersed cervix may mask the variety of phenotypes that cohabitate within each subregion or the vascular compartment. Variations in cell nuclei density/area, the possible incision of adherent extra-cervix that may not be trimmed prior to processing in some, but not all tissues, and the imprecise boundary between cervix and uterus in gross dissection, are not insurmountable considerations for understanding local functions of macrophages. In other respects, the challenge to co-localize mature macrophages and identify specific phenotypes to distinguish inflammatory from anti-inflammatory activity has had some success using flow cytometry, but not *in situ* with immunohistological approaches using surface or intracellular markers for resident immune cells. Other methods are needed to determine, with certainty, the local phenotypic activities or the consequence of intercellular interactions between fibroblasts and immune cells in the stroma of the prepartum cervix.

Overall, these findings raise the possibility that some balance of immune cell products guides extracellular remodeling to promote softening, ripening, and the capability to dilate. Retreating to a previous consideration, caution about current understandings of the remodeling process in women may be derived from comparisons of biomolecular data from biopsies without histological confirmation of cervix at unspecified periods at term, before or after labor, or from non-pregnant individuals. In rodents, more biomarkers are needed to fill gaps in understanding the transition from soft to ripening, as well as from ripe to dilation at term to understand pathological processes associated with models of preterm birth. Collectively, the transitions between phases of remodeling do not have sufficient time points to resolve the essential biomarkers to track ripening or its completion to predict the capability for the cervix to dilate whether at term or the premature loss of competency as occurs in some women at risk for preterm birth (113, 114). Thus, far, at least some cellular and structural biomarkers have been identified to track the progression of remodeling during the transition from a soft to ripe cervix. Studies of key inflammatory pathways in the intact cervix during pregnancy provide the foundation to fill several major gaps and identify additional biomarkers for the ripening process.

## Schema for Cervix Transformation From Soft to Ripe to Dilation in Parturition

**Figure 3:**
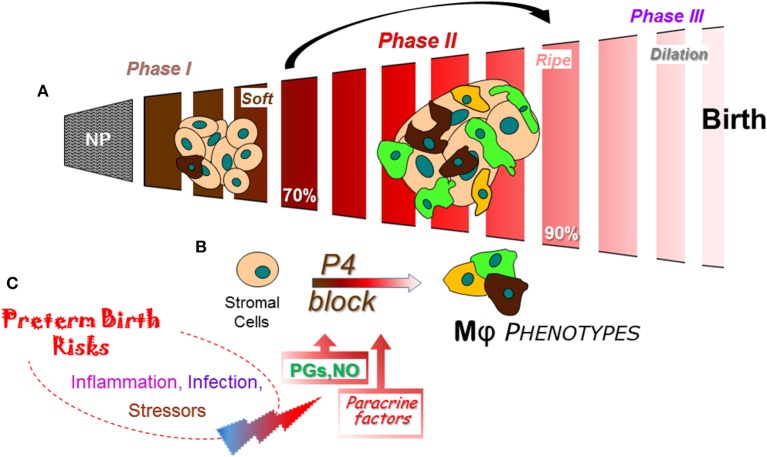
Schema of a final common pathway for phases of cervix remodeling for parturition based on evidence in rodent models with insights from findings in prepartum women (47). **(A)** Morphological structure changes in the cervix during the transition from dense barrier when non-pregnant to soft (Phase I) then ripe (Phase II) that involve degradation of cross-linked collagen and reduced cell nuclei density. Evidence indicates that the transition from Phase I to II may be driven by increased density of macrophages (Mϕs) and diverse phenotypic activities (different colors). NP, non-pregnant. Percentage (%) of pregnancy based upon day or week of pregnancy/average term birth (19–20 days for mice; 22–23 days for rats) or 40 weeks for women. **(B)** Proposed cross talk between stromal fibroblasts with progesterone receptors that mediate trophic effects of this hormone and Mϕs that produce a variety of paracrine factors that drive a functional progesterone withdrawal before 85% of pregnancy., Paracrine guidance for functional activities of stroma cells and macrophages may include prostaglandin (PGF2α), nitric oxide (NO), proinflammatory and phagocyte-related cytokines, chemokines, hypoxia-linked molecules, and VEGF to advance extracellular collagen matrix degradation and loss of barrier properties that enhance distensibility. This process ultimately leads to a virtual disappearance of the cervix for dilation (Phase III) in temporal coincidence with labor before birth. **(C)** Convergence of inflammatory stimuli and risk factors are conceived to regulate the timing and pace of premature cervix remodeling. A variety of coincident proinflammatory stimuli (bacterial or viral biome in vagina), pathophysiological considerations (prior sensitivities), or genetic susceptibilities, may ultimately tip the balance away from local anti-inflammatory processes and prostaglandin metabolism to exceed a threshold that accelerates cervix remodeling and leads to preterm birth.

In the path to birth at term, expression of key components of an inflammasome characterize the drive for spontaneous labor. Most women have no signs of infection in reproductive tract structures related to pregnancy, thus spontaneous term labor may be considered a physiologic sterile inflammation. This process appears driven by molecules that were the focus of an exquisite study of chorionic membranes from women at term, not in or after labor (115). The premise of the working hypothesis is that with the progression of pregnancy to term a synchronize sequence necessitates cervix ripening to be sufficiently complete for achieve the capability to dilate such that labor can effectively lead to vaginal birth (47). Recent reviews indicate that completion of remodeling occurs before term or is forestalled in ways that require medical interventions for the well-being of the neonate and/or mother (48, 116, 117). The summative effects of various risk factors are conceived to exceed a threshold for physiological inflammation to promote the transition from soft to ripening in the mechanism for parturition (Figure 3 Schema). Thus, the premise of the working hypothesis is that labor begins after cervix ripening is completed.

From a restrictive and rigid barrier before pregnancy (Figure 3A), the cervix grows and softens during Phase 1 of remodeling under the trophic influences of a variety of hormones and ovarian steroids (47). Changes in the census of some immune cells are indicated, but replicable studies are needed with frequent time points during the period from early pregnancy into the 2nd trimester. A similar gap during the Phase 2 transition from a soft to ripe cervix is bridged with conclusions from findings in a soft compared to ripe cervix, i.e., before and after ripening. The current consensus is that fibrillary collagen in the extracellular matrix of the stroma is gradually replaced with less cross-linked collagen during the transition from a soft to ripe cervix. However, as discussed above, a histological study of the cervix in mice during the transition to Phase II indicates an increase in resident macrophages other structural characteristics by Phase III dilation and birth (39). The temporal relationship of these dynamic changes in cervix physiology with a functional loss of progesterone actions for remodeling in rodents and humans has only recently been appreciated. Dramatic differences in density of macrophages and diversity in their morphological shape raise the possibility for an increased assortment of phenotypes and immune cell activities before the shift to Phase III Dilation. The cellular, structural, and biomolecular characteristics described above reflect an emerging definition for ripening- a physiological inflammation to drive cervix remodeling and the capability to dilate. For the proposed mechanism that regulates ripening (Figure 3B), a cross talk between cells in the stroma is proposed to regulate local responsiveness to progesterone and inflammation by resident immune cells. In mice and rats, fibroblasts in the cervix stroma are the predominant cell with classic genomic PR (83, 87, 118). Sparse distribution of PR cells in the luminal epithelium notwithstanding, there is little evidence to suggest resident macrophages or other cells have PR. Moreover, only the PR-A, not PR-B isoform is necessary to mediate effects of progesterone for all reproductive functions (84, 118). In the absence of information about membrane PR in the cervix, the current concept is that stromal fibroblasts integrate various convergent local factors and systemic influences to mediate progesterone efficacy. Paracrine signals from fibroblasts then regulate resident macrophage functions and, in turn, guide the progression of subsequent stromal cell activities for extracellular matrix remodeling.

As part of the stroma-immune cell cross talk during ripening, paracrine factors may include increased hyaluronic acid (32), nitric oxide production (119), greater prostaglandin actions through reduced enzymatic degradation (120), and proinflammatory mediators (121) to regulate extracellular structure, local blood flow, and enhance vascular permeability (Figure 3C). How these signals may coalesce to regulate the variety of immune cell phenotypes that are present in the prepartum cervix with the potential for opposing functions is not known. Indeed, appreciable amounts of mRNAs and proteins for macrophage- and lymphoid-related cytokines with classic opposing activities are detected in the same biopsy from cervix or lower uterine segment before term and with preterm birth (42, 89, 93, 100, 122, 123). In a comprehensive review of the immunology of parturition (124), factors that include IL-1β can act on a number of cell types to increase the production of cyclooxygenase (COX)-2 and prostaglandin E2 to facilitate cervical dilation in women. IL-1α, which uses the same receptor as IL-1β, has been shown to increase COX-2 and PGE2 production by rabbit cervical smooth muscle cells and fibroblasts, regulate increase release of local proteinases, and may indirectly increase permeability of blood vessels for leukocyte trafficking. Of importance to note, as previously mentioned, macrophages have the capabilities to produce nitric oxide and prostaglandins. Inhibition of nitric oxide or prostaglandins synthesis suppresses cervix softening and ripening while stimulation of nitric oxide production or prostaglandin treatment advance remodeling and induce preterm birth in rats (125), mice (83, 126), and women (127). These findings collectively support the conclusion that nitric oxide and prostaglandins are a critical part of the common mechanism for birth at term and with preterm birth.

The role of T lymphocytes in remodeling has yet to be studied in the prepartum cervix. As described earlier, limited presence of lymphoid-derived cells in the cervix, as well as clustering in subepithelial and luminal regions, suggest a limited role in the stroma. Moreover, immunosuppression therapy that blocks adaptive immune responses in transplant patients does not interfere with pregnancy (128, 129). Effects of immunosuppression on parturition are not known since most women with solid organ transplants are delivered by Cesarean section (130). Thus, current evidence focuses on macrophages to mediate inflammation in the structural remodeling process as informed by paracrine factors produced by stromal fibroblasts that integrate progesterone and other signals at the external fetal-maternal interface.

## Immunobiologic Perspective on Cervix Remodeling With Preterm Birth

Worldwide, perinatal morbidity is most associated with preterm birth before 37 weeks of pregnancy. A classic review categorizes three major causes of preterm birth (131), (1) about half are spontaneous of unknown etiology, a portion of which include premature rupture of fetal membranes; (2) nearly a quarter represent maternal or fetal complications that include hypertension, hemorrhage, or intrauterine growth restriction; and (3) the remaining quarter have multiple pregnancy, cervix incompetence, or uterine-placental malformations. Another perspective indicates that nearly a third of women with preterm birth have infections that involve the maternal reproductive tract or fetus (132). While opening of the cervix represents the unlocking of the gate for birth at term, reports about preterm birth rarely include information about cervix remodeling—a consequence of the preterm labor emergency with the cervix partially or completely dilated. Whether the fetus is alive or not, fetal demise is not necessarily associated with cervix remodeling and vaginal delivery (133). Well before 37 weeks of pregnancy, premature newborns can be a fraction of the weight and size of babies at term. The same is true for pups from mice induced to give birth preterm about days 15–17 of pregnancy vs. those at term on days 19–20 postbreeding (106, 134). These observations about newborn size difference, irrespective of etiology for preterm labor, raise the possibility that inflammatory processes that drive ripening, compliance, and dilation may not necessarily need to be the same in preterm vs. term birth.

Although spontaneous term labor may be considered a state of physiologic sterile inflammation, most women at term have no signs of intra-amniotic or other infection. Key components of this process have been described as an inflammasome, a focus of an exquisite study of chorionic membranes from women at term, not in or after labor (115). Another recent outstanding review focused on the role of T cells and macrophages in preterm birth (135). For obvious reasons related to availability of biopsies before preterm birth, less is known about the immunologic correlates associated with risk factors that advance the transition from soft to ripe cervix (48, 116). However, one study of immune cells obtained from the external os of the ectocervix lumen of women with recurrent preterm birth fewer CD14+ macrophages were evident (136). No differences in T or B lymphocytes, NK cells, or several activated phenotypes were found in cervix swabs from women whom delivered early or at term. Whether these findings provide insights about the external fetal-maternal interface at the luminal-epithelial margin or stroma requires further investigation.

By contrast, comparison of preterm and term prepartum cervix remodeling has been studied in several rodent models and women. For regulation of the timing of birth in mice, effects of PR antagonists to induce preterm birth or agonists to block preterm birth are consistent with the working hypothesis in the Figure 3 schema to predictably advance or forestall, respectively, characteristics of cervix remodeling (47). In women given a progesterone receptor antagonist to terminate a first trimester pregnancy, cervix dilatation occurred within 16 h without a change in collagen (hydroxyproline concentration) in a biopsy from the ectocervix (43). Moreover, a comparable study found that progesterone antagonist markedly increased, within 24 h, tissue resident macrophages, and neutrophils in the cervix (137). Increased abundance of abundance of monocyte chemotactic protein-1, MMP-8 (neutrophil collagenase), and prostaglandins were also evident. IL-8 synthesis by cervical fibroblasts (138, 139) was inhibited by progesterone and this block could be mediated by the transcription factor NFkB, which is also regulated by progesterone (140). Within the context of the current working hypothesis (Figure 3), these findings support the possibility that PR modulator effects on stromal fibroblasts guide actions by macrophages to regulate local inflammation in the cervix.

Treatments to induce inflammation are a common model for preterm birth. Intrauterine or intraperitoneal injection with the bacterial endotoxin lipopolysaccharide (LPS) induces preterm prepartum cervix remodeling (141). In some studies, conclusions that different mechanisms mediate the effects of inflammation and withdrawal of progesterone effects on the cervix are premature because interpretations of findings can be complicated by experimental design differences. For instance in two studies in mice, times and day for treatment during pregnancy, as well as latency to obtain cervix for analyses were different (142, 143). Whether results about cervix remodeling are even comparable at 6, 1, or 12 h before birth in controls (day 18.5 of pregnancy), or after LPS or progesterone antagonist-induced preterm birth, respectively, is not known. Replication of results, with frequent time points and contemporary vehicle-treated controls before appearance of the first pup are needed to advance understanding of the remodeling process. In addition, concerns about the usefulness of the intrauterine administration of LPS as a model for ascending infection-induced preterm birth in women were raised by a compelling study that found systemic, but not intravaginal treatment induce preterm birth in mice (141). Except for introduction of LPS into the endocervix (144), which breaches the luminal epithelial immune interface, there is consistent evidence for systemic inflammatory drive of preterm birth. By example, the importance of myeloid-derived immune cells and activities for cervix remodeling was emphasized in a recent study in mice that used antibody treatment to suppress the cytokine granulocyte-macrophage colony stimulating factor (GM-CSF) and block LPS-induced preterm birth (145). GM-CSF acts to reduces myeloid cell migrations, stimulates their differentiation, and proinflammatory activities (146). With relevance for women at high risk for preterm birth as well, findings indicate that inflammatory proteins increased in amniotic fluid before premature delivery (123). Thus, a more intensive study of the prepartum cervix in these rodent models during the interval between treatment and preterm birth may identify biomolecular markers that have predictive comparative relevance for impending preterm birth or treatments that forestall advances in cervix remodeling.

The common use of mice to investigate inflammation-induced preterm parturition has also served as a model to study the effects of a variety of agents to block preterm birth. By example, the effects of LPS to induce preterm birth can be blocked by systemic or intravaginal treatments with a variety of anti-inflammatory agents (145, 147, 148). Although effects of these or related compounds on cervix remodeling have yet to be studied, the implication is that, like GM-CSF, actions may be upon upstream mechanisms related to myeloid cell phenotypic actions in the final common pathway for parturition. By contrast, a recent novel discovery indicates that intravaginal Replens prevents breakdown of the cervical epithelial barrier and preterm birth induced by i.u. LPS through a mechanism that involves reduced interferon-mediated upregulation of MMP13 and degradation of the cell adhesion protein E-Cadherin (149). LPS is indicated increase permeability through an action on cervical epithelial cells from the ectocervix and endocervix (121). Conceivably, disruption of the cervical mucus/hyaluronic barrier in cervix subregions may breach the external interface to allow entry of pathogens and increase risks for preterm birth. These findings supports an additional possibility that degradation of the luminal-epithelium barrier by intrauterine inflammation may directly contribute to the advance in collagen degradation and reduced density of cell nuclei in the stroma as part of premature cervix ripening before preterm birth.

Beyond the scope of this review is the nuanced controversy about whether progesterone is effective for treatment of preterm birth or childhood consequences. Much has been written on this topic (150–153), though far less is known about consequences on the characteristics of remodeling or immune cells in the cervix. In mice, progesterone or progestational agents clearly regulate inflammatory characteristics associated with cervix remodeling (Figure 2 as discussed earlier). Since LPS suppresses serum progesterone in pregnant mice, are LPS actions mediated by inflammation alone or systemic withdrawal of progesterone? Except for one study that administered LPS i.p. to mice early in pregnancy (154), progesterone does not block preterm birth induced by i.u. or i.p. LPS treatment (155, 156). In one respect, birth is a complex compound endpoint for efficacy of progesterone treatment in women at risk and for experimental models for preterm birth. Perhaps reconciliation of discrepant conclusions might result from a consensus to assess other biomarkers related to cervix remodeling that track progesterone actions at the physiological, structural, cellular, or molecular levels. By example, do various progesterone treatments actually affect the cervix with respect to increase local concentrations of the hormone, alter mucus secretion, reduce remodeling characteristics, suppress inflammasome components, or proinflammatory molecules? In addition, such biomarkers and current endpoints for inflammation provide an opportunity to explore why cerclage, pessaries, and bed rest appear to reduce the incidence of preterm birth in women (157, 158). Development of biomarkers for critical intersections in the remodeling process may benefit the search for novel therapeutic approaches to regulate ripening of the cervix, both before or at term. Major questions in current clinical practice may also be addressed, for instance why does preterm birth not result in all women who present with preterm labor?

## Immunological Focus Associated With Cervix Remodeling Complications and Delayed Birth

There is little to review about the status of immune cells or processes in the cervix of any species with when pregnancy extends beyond term. Whether remodeling is forestalled or inflammation suppressed in the cervix when birth does not occur at term is not known.

Insights gleaned from mouse models in which genes are altered or knocked out have proven useful for focus on important molecules for cervix remodeling. Two of a number of examples in a previous review are informative for the question of whether there is an immune contribution to delayed birth and forestalled cervix remodeling (47). First, the prostaglandin F2α receptor is essential for ripening of the cervix (83). Mice lacking this receptor, get pregnant, but do not deliver. All characteristics associates with softening and the initial transition to ripening are the same up until the day before birth when fewer macrophages and less cross linked collagen distinguish knockouts from wild-type controls. In coincidence with increased density of macrophages and degradation of collagen, progesterone withdrawal after ovariectomy induces birth of live pups within 24 h. These finding support the link between enhanced residence and activities by macrophages in association with ripening and the capability of the cervix to dilate for birth. Second, in mice lacking the steroid 5a-reductase type 1 enzyme (*5aR1*^−/−^), up to two-thirds of pregnant females depending upon background strain fail to deliver at term (159). Investigation of the parturition defect found that between days 15–17 of pregnancy, there were no differences in wild-type vs. *5aR1*^−/−^mice in biomechanical properties of cervix compliance; serum and cervix concentrations of progesterone or the 5α reductase product, 20a-hydroxyprogesterone were also equivalent. Rather than support the conclusion that progesterone catabolism plays any role in the parturition defect in this mutant mouse or in cervix remodeling at term given major remodeling in cervix prior to day 17 of pregnancy, attention focuses on the uterus where progesterone concentrations are sustained at peak of pregnancy and differences in catabolism of progesterone to 20a-hydroxyprogesterone fails to occur in *5aR1*^−/−^mice. Finally, the importance of changes in relaxin or decorin (160), as well as nitric oxide production by inducible nitric oxide synthase (161) for cervix remodeling and parturition has been questioned because knockout rodent models indicate these molecules are not necessary for birth of live pups and parturition at term.

What is known is that fetuses beyond 39 weeks of gestation require medical intervention for delivery. Prenatal growth may exceed the capabilities of maternal placental function and for passage through the birth canal. Potential complication for both the newborn and mother challenge modern clinical practice as to whether to deliver, facilitate, or wait for the start of parturition. Induction of labor (IOL) has increased more than 5-fold since Bishop first proposed a scoring system to predict successful induction of vaginal delivery for women at term with prior history of vaginal birth (162) and more than doubled in the last 2 decades to exceed 25% of all births following guidelines recommended by the American College of Obstetricians and Gynecologists (77). Maternal benefits and improved well-being for neonates are clear (163). The question of the best methods for IOL was recently reviewed (164). Among the pharmacologic methods considered for IOL and to ripen the cervix are administration of prostaglandins and nitric oxide. These agents have been featured to be involved in proinflammatory activities that link macrophage-related products and stromal cervical fibroblast cross-talk with cervix remodeling. Mechanical methods, some centuries old and still in use were also discussed. However, no one method was determined to be superior to ripen the cervix. In fact, there is no method validated to reflect a biomolecular or morphological characteristic of remodeling to determine cervix favorability for vaginal delivery. Assessment of the condition of the cervix is important because, if the goal of IOL is vaginal delivery within 18 h then IOL failed in more than a third of the obstetrical cases (165). As noted, “Cervical ripening by physical or pharmacological methods and labor induction should not be confused, even though the literature usually refers to labor induction as the process also including cervical ripening.” This statement represent a major take home message of this review, i.e., ripening is distinct from and its completion coincides with the capability of the ripened cervix to dilate in phase 3 of remodeling. These realizations now present an important opportunity to investigate the effects of several of pharmacologic and mechanical methods for IOL to regulate characteristics of inflammation at critical waypoints in the path to remodeling from IOL to vaginal delivery compared to failed IOL. Such efforts have translational potential to develop a novel immunotherapeutic approach to complete cervix ripening and facilitate success of IOL.

## Prospects for Non-Invasive Approaches to Assess Characteristics of Inflammation In Cervix

Multiphoton microscopy applies fluorescence technology to imaging structures in thick sections and live tissues. Second harmonic generation (SHG) is the most popular version of this technique with strength and challenges that limit usefulness to assess cervix remodeling during pregnancy (166, 167). By contrast, ultrasound is commonly used for diagnostic imaging in obstetrical practice. In fact, sonography was used to determine that a short cervix of <25 mm at 16–24 weeks of gestation is the strongest clinical risk factor for preterm birth (168). For a variety of reasons, several reviews concluded that there is insufficient evidence to recommend transvaginal ultrasound of cervical length as clinically useful to predict preterm birth in pregnant women with singleton or twin gestations (169, 170). However, the benefits of developments in technology and analyses were evident in recent study in which quantitative ultrasound analyses was able to distinguish a reduction in microstructure of the cervix in women that were early in pregnancy or at term (5–14 vs. 37–41 weeks, respectively) (171). The improved resolution resulted for use of a backscattered power parameter estimation to reduce signal variability due to anisotropy (echo from complexity of fibrillary network) and spatial heterogeneity in tissue. Whether this approach can be validated to assess a biomolecular or biomechanical characteristic of inflammation or collagen degradation during remodeling or have the sensitivity to distinguish the transition from a soft to ripening cervix remains to be determined. Of importance to mention for the remodeling process is the potential benefit for *in vivo* assessment of biomechanical capabilities of the fetal membrane and cervix through application of a finite element model that was based upon MRI evaluation of a patient with a normal vs. short length (172). Thus, it is premature to discount the potential of transvaginal ultrasound to assess cervix remodeling in a longitudinal study of prepartum women at term or at risk for preterm birth.

Another approach is to use Raman spectroscopy to monitor the biochemical makeup and molecule concentration based upon signal intensity of the cervix *in vivo* throughout pregnancy. In a recent longitudinal study of women during pregnancy, this approach proved useful to identify many peaks that reflected extracellular matrix proteins, actin, and blood in the cervix that change throughout pregnancy and postpartum (173). This approach has also been used to study changes in fatty acid lipids, proteins, and amino acids the cervix in mice during pregnancy (174), as well as water content in non-pregnant and mice at term (175). Whether clear resolution of potentially overlapping peaks that correspond to signatures of known molecules in the inflammatory process of remodeling awaits further study.

Thus, developments in non-invasive technologies to image molecules and cells that are relevant for inflammation is an objective of ongoing investigation. The goal is to validate and analysis of biomarkers that characterize the transition between phases of remodeling leading up to birth at term. Subsequent application of this approach would help test hypotheses about the timing and extent of ripening before preterm birth. This information would be valuable to distinguish inflammation from infection and address many unanswered question about the mechanism through which risk factors contribute to the advance of cervix ripening in preterm birth or inadequate remodeling in women that forecast difficulties with parturition.

## Conclusions

For the mechanism of parturition, the prevailing perspective has been that sterile intrauterine inflammation drives a massive release of proinflammatory cytokines and an influx of maternal leukocytes, to initiate labor. At some point along this path, the cervix ripens and dilates with labor for birth to occur. Evidence has accumulated to suggest that less is known about the temporal sequence of events that coordinate a symphony of functions by various reproductive structures at the internal fetal-maternal interface and for the purpose of this review, in a portion of the external fetal-maternal interface in the cervix, to open the gate for birth. Over that past several years, this review provides a summary to indicate that the previous prevailing perspective is predominantly based upon observations among available gestational tissues across species from before labor in comparison to during or after delivery. Details about regional specificity within distinct reproductive organs, subregions of the cervix in particular, and generalizations to bridge temporal gaps in data are now recognized to have provided limited insights into the essential sequence of changes that lead to spontaneous labor and birth in the process of parturition. Focus on the importance of a coordinated time course for the 3 phases of cervix remodeling, involve activities by immune cells (principally myeloid-derived cells, for now) and guidance by local paracrine signals, at a time much earlier than previously thought. This perspective needs to be expanded to understand phenotypic activities by macrophages and other immune cells of lymphoid lineage in the cervix. Moreover, the time course of critical cellular and biomolecular processes in fetal membranes of the so-called zone of altered morphology that overlays the internal os of the cervix (176), as well as within the internal fetal-maternal interface must be integrated to develop a unified mechanism for parturition. Hypotheses generated and tested in various animal models described in this review would help identify the chronology of relevant biomarkers for translational studies and with clinical relevance. Looking ahead, enthusiasm has returned to a long-standing theory that the fetal allograft informs the maternal host of readiness for life outside the womb. Whether fetal signals promote local inflammation directly, by export of gestation specific exosomes (177), or indirectly, through components of the internal or external fetal-maternal interfaces, is conceived to unleash an effective, but limited local graft-like rejection sequence for parturition to benefit neonate well-being, as well as maternal healing for caretaking of the newborn and subsequent reproductive activity.

## Author Contributions

SY conceived, wrote, and created the figures for this manuscript.

### Conflict of Interest

The author declares that the research was conducted in the absence of any commercial or financial relationships that could be construed as a potential conflict of interest.
